# Activation of autophagy reverses gemcitabine-induced immune inhibition of RAW264.7 macrophages by promoting TNF-α, IL-6 and MHC-II expression

**DOI:** 10.1007/s12026-021-09210-7

**Published:** 2021-07-14

**Authors:** Shanshan Jiang, Rong Wang, Lu Han, Kudelaidi Kuerban, Li Ye, Shu Pan, Shengnan Li, Yongfang Yuan

**Affiliations:** 1 Department of Pharmacy, Shanghai Ninth People’s Hospital, Shanghai JiaoTong University School of Medicine, Shanghai, 200011 China; 2 Department of Microbiological and Biochemical Pharmacy & The Key Laboratory of Smart Drug Delivery, Ministry of Education, School of Pharmacy, Fudan University, Shanghai, 201203 China

**Keywords:** RAW264.7 macrophages, Immune inhibition, Autophagy, Inflammatory cytokines

## Abstract

This research aims to investigate the effect of gemcitabine (GEM) on various activities and functions of macrophages. Phagocytosis, cell autophagy and reactive oxygen species (ROS) were analysed by laser scanning confocal microscope. The cell cycle status and major histocompatibility complex II (MHC-II) expression were examined by flow cytometry. Inflammatory cytokine secretion such as tumour necrosis factor α (TNF-α) and interleukin 6 (IL-6) was detected by Elisa assay. The expression of proteins was analysed by western blot method. The results revealed that GEM-induced immune inhibition of M1-type RAW264.7 macrophages activated by interferon-γ (IFN-γ) and lipopolysaccharide (LPS). We also found that GEM inhibited autophagy, as evidenced by the reduced formation of autophagosome-like vacuoles and autophagosomes. Further study showed that incubation of activated macrophages with the autophagy inhibitor 3-MA induced immune suppression. In contrast, treatment with the autophagy inducer trehalose (Tre) restored phagocytosis, TNF-α and IL-6 secretion, and MHC-II expression in GEM-induced immune-inhibited macrophages. GEM reduced immune effect of M1-type RAW264.7 macrophages via inhibiting TNF-α, IL-6 and MHC-II expression. Furthermore, activation of autophagy by Tre reversed GEM-induced immune inhibition of RAW264.7 macrophages.

**Supplementary Information:**

The online version contains supplementary material available at 10.1007/s12026-021-09210-7.

## Introduction

Gemcitabine (2′,-2′-difluoro-deoxycytidine, GEM) is a first-line treatment for pancreatic cancer. However, its efficacy is limited by the acquisition of drug resistance after long-term use [1]. In addition to its cytotoxic effect, GEM has been shown to exert immunomodulatory activity in different animal tumour models. GEM has been confirmed to selectively deplete regulatory T cells, myeloid-derived suppressor cells and B lymphocytes in tumour-bearing models [2, 3].

Macrophage-based immunotherapies for cancer are more effective and tolerable than radiotherapeutics, chemotherapy or surgery [4]. Macrophages are the most abundant immune cells, comprising 5–40% of the total mass in solid tumours [5]. Recent trials confirmed that the presence of tumour-associated macrophages (TAMs) has been positively linked with poor prognosis in some cancers such as melanoma and breast cancer [6]. Macrophages are highly plastic cells that can be ‘classically activated’ to the pro-inflammatory (M1) phenotype in early tumours and exert tumouricidal effects [7]. Whereas in established tumours, macrophages tend to be ‘alternatively activated’ to the immunosuppressive (M2) phenotype and promote tumour progression [8]. In response to an activating stimulus M1 polarization predominates, follows to release of pro-inflammatory cytokines such as reactive oxygen species (ROS) and nitric oxide (NO). However, increased M2 polarization subsequently mediates resolution of the inflammation [9]. M1 macrophages are characterized by their high capacity of phagocytosis, inflammatory cytokine secretion and free radical production [10, 11]. TAMs are a crucial target for anti-tumour treatment. Attempts to increase the M1/M2 ratio by inducing TAMs to switch from the M2 to M1 phenotype effectively reduced tumour malignancy in vivo [12]. Reprogram TAMs into a pro-inflammatory M1 profile contributed the anti-tumour efficacy of hydrazinocurcumin [13]. However, the effect of GEM on M1-type macrophages was unclear. In this study, we hypothesized that GEM might affect the function of M1 macrophages by altering their activity.

To confirm this hypothesis, macrophages were incubated with the Th1 cytokines IFN-γ and LPS, which polarize macrophages to the pro-inflammatory, classically activated, M1 phenotype [14], with or without GEM, and their phenotype and functions were assessed. Interestingly, we discovered that GEM treatment of IFN-γ/LPS-activated M1 macrophages not only suppressed phagocytosis but also influenced autophagy. Autophagy regulates numerous cellular processes, such as degradation of dysfunctional or unnecessary intracellular components in many cell types [15]. It is necessary to conceive potential strategies aimed at targeting mechanisms involved in macrophage activation.

Autophagy has been widely studied in immune cells, such as dendritic cells, B cells, T cells and macrophages, and these studies showed that autophagy plays important roles in regulation of inflammation-modulating functions [16]. Song et al. revealed that activation of autophagy could overcome asparaginase-induced immune suppression in M1 macrophages [14]. The impaired of macrophage autophagy could aggravate liver damage by accelerating M1-type macrophage polarization in the model of hepatic steatosis [17]. The emerging recognition concerning autophagy regulates immune responses suggested that GEM-induced changes in macrophage autophagy could be a mechanism underlying the immune inhibition which marks this condition. These researches prompted us to investigate the function of autophagy in GEM-induced regulation of macrophages.

In the manuscript, we demonstrate that GEM induces immune suppression as well as autophagy of M1-type RAW264.7 macrophages via inhibiting phagocytosis, cytokine secretion and MHC-II production. Further study elucidates that activating autophagy with trehalose (an autophagy inducer, Tre) reverses GEM-induced immune inhibition of M1 macrophages. These findings advance our understanding of the effects of GEM on M1-activated macrophages and propose new insight into the role of autophagy in GEM-induced macrophage inhibition.

## Materials and methods

### Materials

Gemcitabine hydrochloride was obtained from Dalian Meilun Biotech Co., Ltd (Dalian, China). Cell cycle detection and CFDASE cell proliferation and tracking kits were obtained from KeyGen BioTECH (Nanjing, China). 3-MA, IFN-γ and LPS were purchased from Sigma-Aldrich (St. Louis, MO, USA). Griess reagent was obtained from Beyotime Institute of Biotechnology. TNF-α, IL-6, IL12 and MCP-1 ELISA kits were purchased from Boatman Biotech (Shanghai, China). Primary antibodies against ATG5, Beclin1, SQSTM1/p62 and LC3B were purchased from abcam company (Cambridge, MA), and MHC-II was purchased from Cell Signaling Technology, Inc., whereas the antibody against actin and horseradish peroxidase-conjugated secondary antibodies against mouse or rabbit IgG were purchased from Weiao Biotechnology Co., Ltd.

### Cell lines and culture

Murine RAW264.7 macrophages were purchased from Cell Bank of Chinese Academy of Sciences. RAW264.7 cells were cultured in RPMI-1640 (Corning Inc.) medium supplemented with 10% foetal bovine serum (Gibco; Thermo Fisher Scientific, Inc.), 100 U/ml of penicillin and 100 μg/ml of streptomycin (Nanjing KeyGen Biotech Co., Ltd.) at 37 °C with 5% CO_2_ in an incubator.

### Macrophage treatment

RAW264.7 cells were treated with 300 IU/ml of IFN-γ and 100 ng/ml of LPS, co-incubated with or without 100 ng/ml of GEM at 37 °C for 24 h and then analyzed as described in the following sections.

### Cell viability assessment

RAW264.7 cells were seeded in 96-well plates at a density of 4 × 10^3^ cells/well. After treated with zymosan for indicated concentrations and times, RAW264.7 cells were incubated with 0.5 mg/ml of MTT at 37 °C for 4 h in dark place. The formazan crystals were dissolved in 100 μl of DMSO, and the optical density was subsequently measured using a microplate reader (BioTek Instruments, Inc.).

### Phagocytosis assay

Cells were cultured in confocal dishes (NEST Biotechnology Co., Ltd., Jiangsu, China) at a density of 1 × 10^5^/ml. RAW264.7 cells were treated as described in ‘Macrophage treatment’, and the cells were incubated with zymosan A BioParticles Alexa Fluor 488 (Thermo Fisher Scientific, Inc.) or FITC-labelled *Escherichia coli* at 37 °C for 2 h. After washing twice with cold phosphate-buffered saline (PBS), the cells were observed under a confocal microscope (LSM710; Carl Zeiss, Oberkochen, Germany).

### Cell cycle analysis

RAW264.7 cells were treated with different concentrations of GEM (0, 25, 50, 100, 200 ng/ml) with or without 300 IU/ml of IFN-γ or 100 ng/ml of LPS for 24 h. The cells were fixed in 70% ethanol at 4 °C for at least 2 h. And then, the cells were washed twice with ice-cold PBS and incubated with RNaseA (Nanjing KeyGen Biotech Co., Ltd) at 37 °C for 30 min. Subsequently, cells were stained with propidium iodide (Nanjing KeyGen Biotech Co., Ltd) at 4 °C for 30 min. FACS Calibur flow cytometry (BD Biosciences) was used to detect the cell cycle status of the cells. The data were analyzed by Flow Jo software version 7.6.1 (TreeStar).

### Transmission electron microscopy (TEM) assays

RAW264.7 cells were treated as described in ‘Macrophage treatment’. After treatment, the cells were harvested and fixed in 2.5% phosphate-buffered glutaraldehyde for TEM (JEOL Co., Ltd., Japan) assay.

### Confocal microscopy assays

RAW264.7 cells were seeded in glass bottom cell culture dishes (NEST Biotechnology Co., Ltd., Jiangsu, China) at a density of 1 × 10^5^/ml and treated as described in ‘Macrophage treatment’. Then, the cells were labelled with the nuclear dye Hoechst 33,342, autophagosome Cyto-ID Green dye, or MitoSox™ red ROS detection dye (ENZO Life Science, Farmingdale, NY, USA) at 37 °C for 15 min. The labelled samples were analyzed using confocal microscope (LSM710; Carl Zeiss, Oberkochen, Germany). The quantitation analysis was performed by ImageJ software version 1.47 (National Institutes of Health).

### NO analysis

RAW264.7 cells were treated as described in ‘Macrophage treatment’. NO generation in cell supernatants was assessed using Griess reagent (Beyotime Institute of Biotechnology, Haimen, China). Briefly, the cells were incubated with equal volumes of Griess reagent I and Griess reagent II for 10 min. Then, the optical density (OD) at a wavelength of 540 nm was analyzed using an ELIASA reader (BioTek Instruments, Inc.). Different concentrations of sodium nitrite (0–100 μM) were used as standards to determine the concentrations of nitrite.

#### Cytokines analysis

RAW264.7 cells were treated as described in ‘Macrophage treatment’. According to the manufacturer’s instructions, cytokine (IL-6, TNF-α) production in the cell supernatants was analyzed using ELISA kits (Boatman Biotech, Shanghai, China).

#### MHC-II expression analysis

Cells were treated as described in the ‘Macrophage treatment’. The macrophages were collected and incubated with antibodies against FCGR2/CD32 and FCGR3/CD16 (FcRγ blocker; BD Biosciences, San Diego, CA, USA) for 5 min. Then, the cells were incubated with a PE-conjugated I-A/I-E antibody for 30 min at 37 °C. The samples were assessed immediately by a FACS Calibur flow cytometry (BD Biosciences).

#### Western blotting assays

Cells were collected and re-suspended in RIPA Cell Lysis Buffer (Nanjing KeyGen Biotech Co., Ltd.) after being treated as described in the ‘Macrophage treatment’. Protein was quantified using the BCA Protein Quantitation kit (Beyotime Institute of Biotechnology, Haimen, China) and equal amounts of total protein (20 μg) were separated by 12% sodium dodecyl sulphate–polyacrylamide gel electrophoresis (SDS-PAGE) and electro-transferred onto polyvinylidene fluoride (PVDF) membranes. The membranes were blocked with 5% (w/v) bovine serum albumin (BSA) in Tris-buffered-saline with Tween-20 (TBST) for 2 h at room temperature, and then, the membranes were incubated with primary antibodies at 4 °C overnight. After washing three times with TBST, the membranes were subjected to peroxidase-conjugated secondary antibodies for 1.5 h at room temperature. Immunoreactive proteins were visualized using an enhanced chemiluminescent detection kit (Pierce; Thermo Fisher Scientific, Inc.). The intensity of immunoreactive bands was quantified by ImageJ version 1.47 (National Institutes of Health).

#### Statistical analysis

All experiments were performed three times. Statistical analysis was performed using GraphPad Prism 5 (GraphPad Software, Inc.). All data are presented as mean ± standard deviations (SD). Comparisons between different sets of data were performed using unpaired Student’s *t* test (2-tailed). The differences among multiple groups were compared using one-way analysis of variance followed by Tukey’s post hoc test. *P* < 0.05 was considered statistically different.

## Results

### GEM inhibited cell cycle and phagocytosis in M1-type RAW264.7 macrophages

**Fig. 1:**
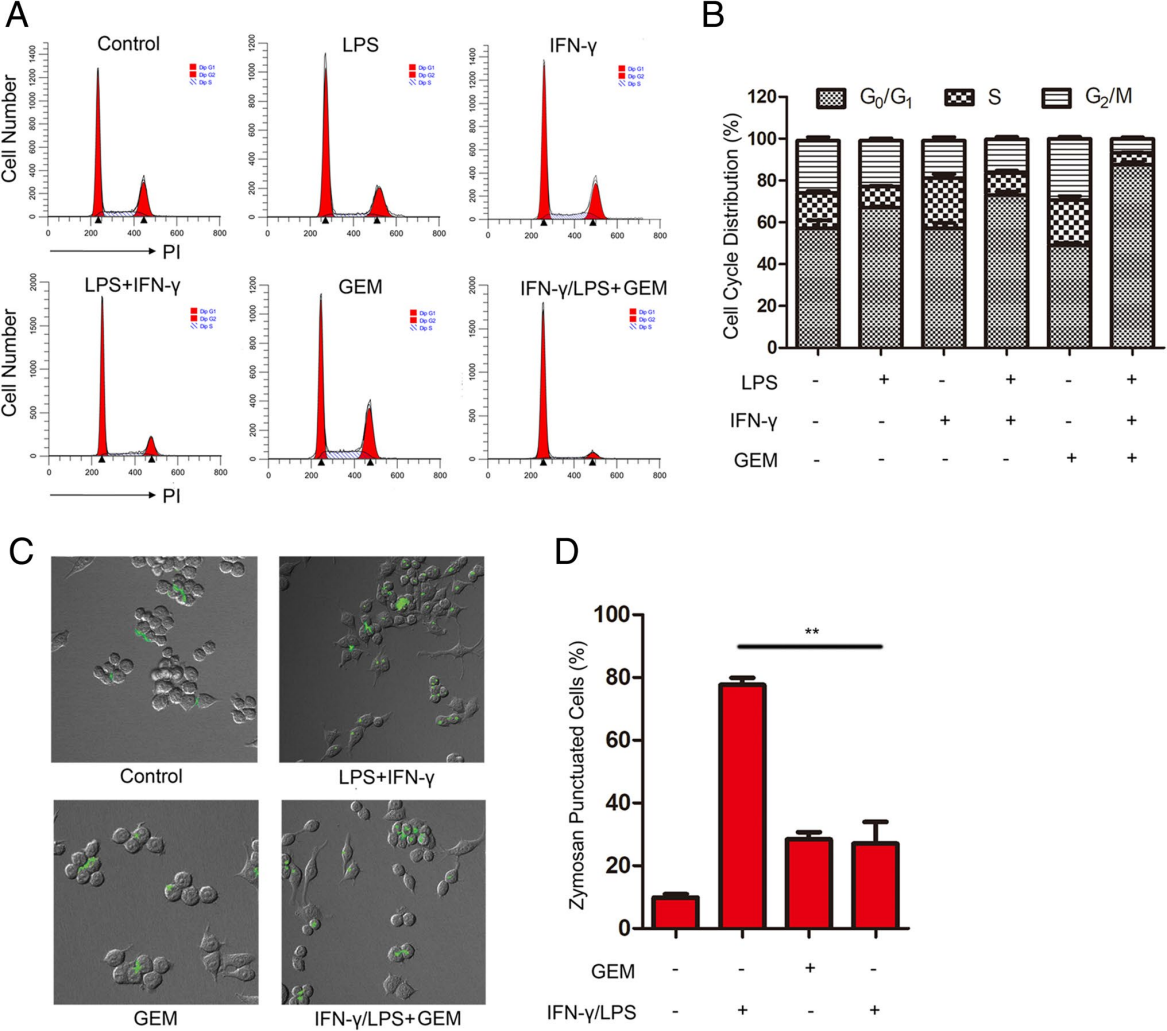
Cell cycle and phagocytosis are inhibited by GEM in RAW264.7 cells. **A** RAW264.7 cells were incubated with 300 IU/ml of IFN-γ or 100 ng/ml of LPS, with or without 100 ng/ml of GEM for 24 h. Cell cycle distribution was examined by flow cytometry analysis. **B** The quantification of cells in different phases of the cell cycle is presented in bar charts. **C**–**D** Cells were treated with 300 IU/ml of IFN-γ and 100 ng/ml of LPS, either alone or in combination with 100 ng/ml of GEM for 24 h. **C** Cells were treated with 2 mg/ml of zymosan particles for another 2 h and the fluorescent punctuation was observed through confocal microscopy. **D** The quantitation analysis of fluorescent spots in Fig. 1C. **P < 0.01

The cell cycle, leading to cell division, consists of a repeating series of events. We first assessed the role of GEM on cell viability and cell cycle in macrophages. The concentration of drugs used in this research did not influence cell viability of macrophages (Fig. S1A-B). IFN-γ and LPS treatment alone did not impact the cell cycle of the macrophages; however, treatment with GEM combined with IFN-γ/LPS led to fewer cells in G_2_/M and S phase and more cells in G_0_/G_1_ phase (Fig. 1A–B). The result indicated that GEM induced G_0_/G_1_-phase arrest which decelerated the cell cycle of M1 macrophages [18].

Phagocytosis is an essential cellular function of macrophages that plays a vital role in innate immunity. Therefore, the effects of GEM on phagocytosis in activated macrophages were assessed by zymosan particles (fluorescent particles which have no cytotoxicity to macrophages in the present experimental conditions) (Fig. S2) and fluorescently labelled bacteria. The results showed that IFN-γ/LPS-treated macrophages displayed obvious green fluorescence of internalized zymosan (*P* < 0.01) (Fig. 1C and D) and FITC-labelled *Escherichia coli DH5α* (*E.coli DH5α*) (Fig. S3), indicating increased phagocytic activity. In contrast, the phagocytosis activity of M1 macrophages was impaired following incubation with GEM. These results demonstrate that GEM might impact the function of M1-activated macrophages.

### GEM inhibited free radical secretion in M1 macrophages

**Fig. 2:**
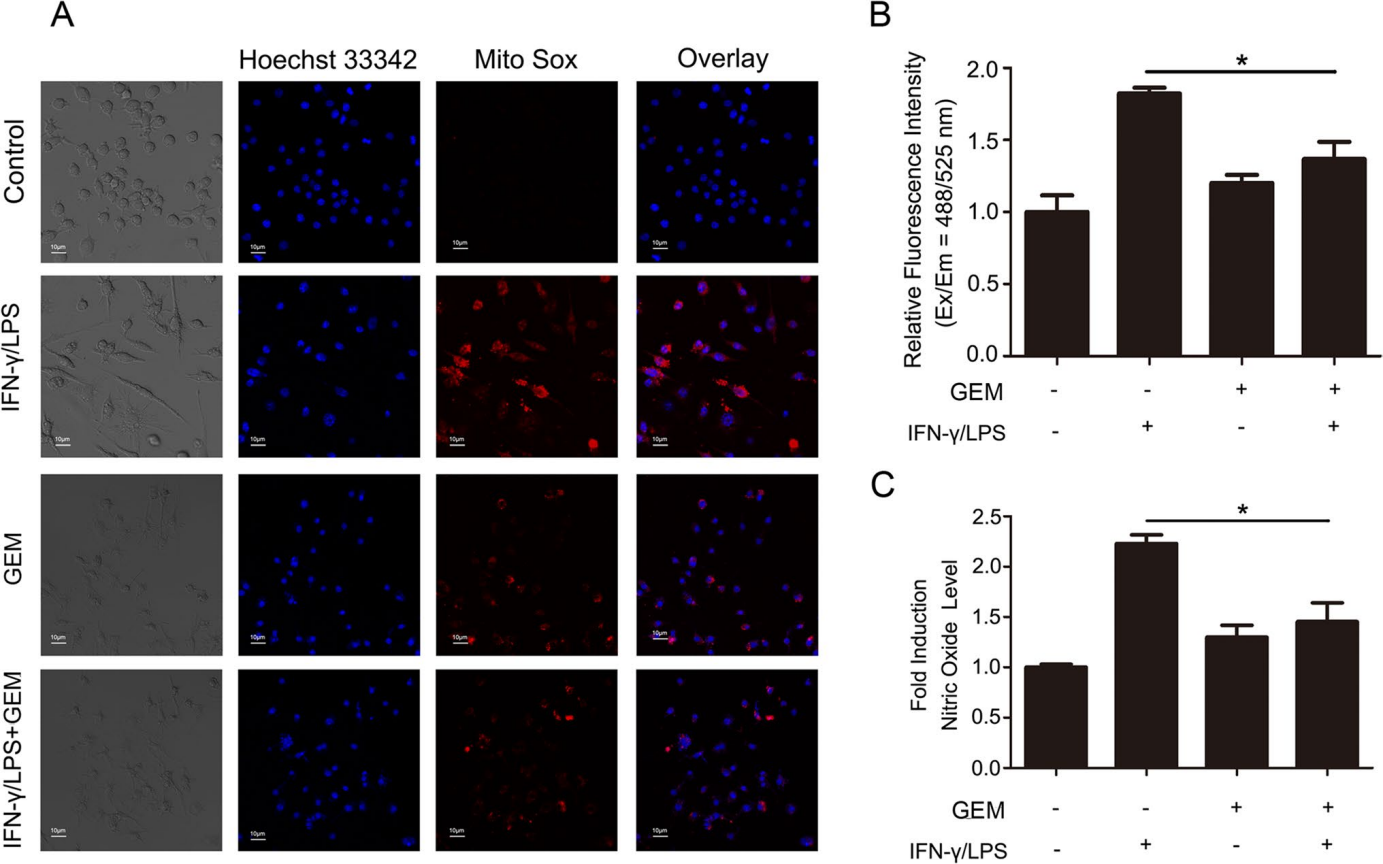
Free radical generation is inhibited by GEM in RAW264.7 cells. **A**–**C** Cells were treated with 300 IU/ml of IFN-γ and 100 ng/ml of LPS, either alone or in combination with 100 ng/ml of GEM for 24 h. A Cells were stained with Mito Sox (an ROS dye) at 37 °C for 15 min and analyzed by confocal fluorescent microscopy. B The relative fluorescent intensity is presented in bar graphs. C The production level of NO was analyzed by Griess reagent, and the OD value was determined by a microplate reader. *P < 0.05

M1-activated macrophages normally secrete high levels of free radicals such as ROS and NO to facilitate the elimination of foreign materials [19]. Herein, we evaluated the ROS and NO levels in GEM-treated macrophages to evaluate whether GEM influences free radical generation in M1 macrophages. As shown in Fig. 2A and B, IFN-γ/LPS-treated macrophages displayed remarkable red fluorescence (*P* < 0.05), indicating a high level of ROS, while the fluorescence intensity of macrophages treated with GEM was lower. NO in RAW264.7 cells was tested by Griess reagent, and NO generation in IFN-γ/LPS-treated RAW264.7 cells was higher than that in control group (*P* < 0.05). In contrast, cells incubated with IFN-γ/LPS and GEM generated less NO than IFN-γ/LPS-treated cells, demonstrating that GEM inhibited NO production in M1 macrophages (Fig. 2C). These data indicate that GEM restrains the generation of ROS and NO in activated macrophages.

### The productions of TNF-α, IL-6 and MHC-II are reduced in GEM-treated M1 macrophages

**Fig. 3:**
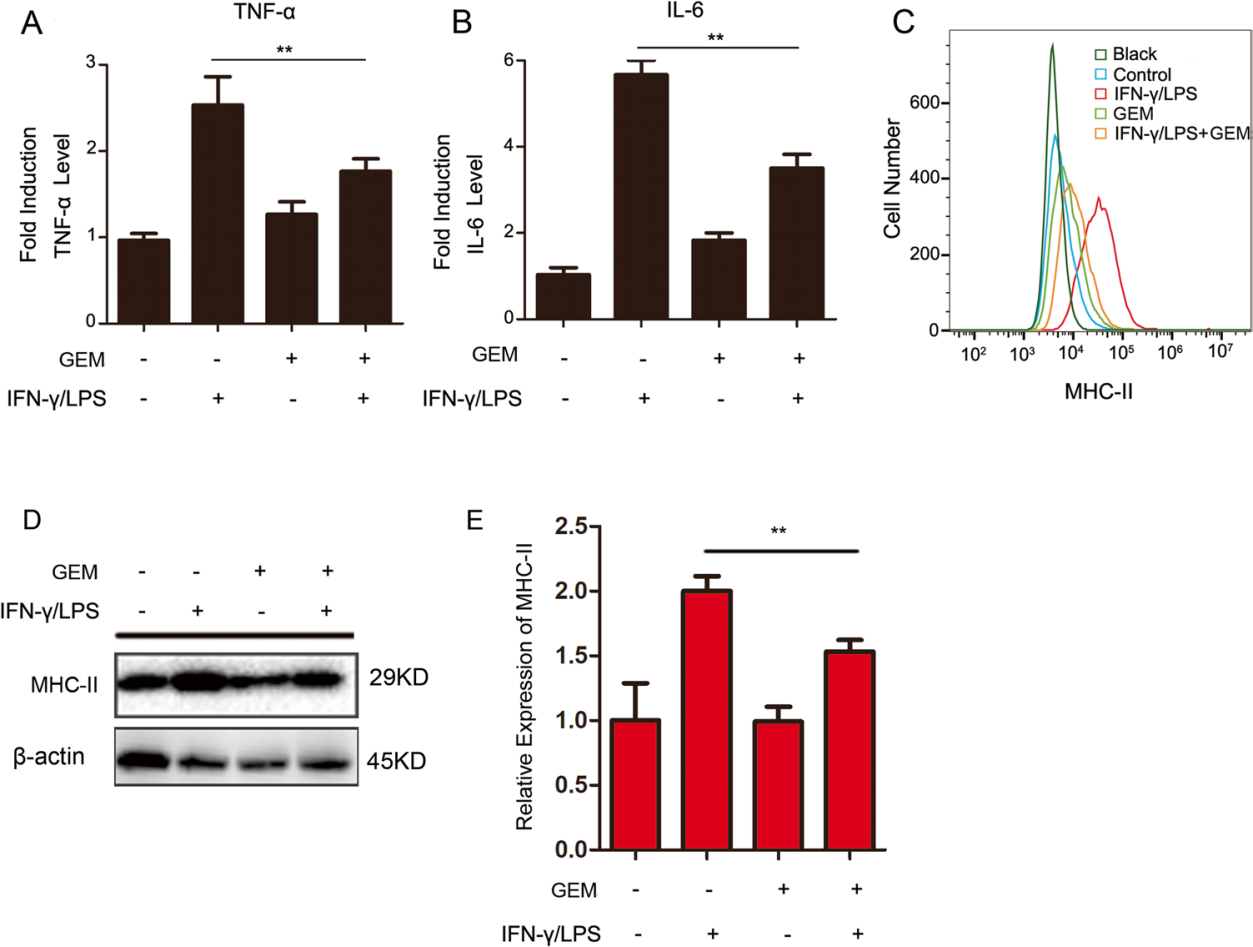
The expression of cytokine and MHC-II is inhibited by GEM in RAW264.7 cells. **A**–**D** Cells were treated with 300 IU/ml of IFN-γ and 100 ng/ml of LPS, either alone or in combination with 100 ng/ml of GEM for 24 h. **A** The content of TNF-α in the cell supernatant was detected by ELISA analysis. **B** The content of IL-6 in the cell supernatant was detected by ELISA analysis. **C** The MHC-II expression was measured by flow cytometry. **D** The expression of MHC-II was examined by western blot analysis. **E** Quantification of MHC-II was presented in bar graphs. **P < 0.01

The generation of pro-inflammatory cytokines is one of the most significant functions of M1-polarized macrophages. Therefore, the impact of GEM on the production of two pro-inflammatory cytokines, TNF-α and IL-6, in M1 macrophages was assessed by ELISA. As shown in Fig. 3A and B, IFN-γ/LPS-treated macrophages showed remarkably increased TNF-α and IL-6 levels, by 2.5- and 5.7-fold, respectively, compared with the levels in untreated cells. TNF-α and IL-6 productions by M1 macrophages were decreased following incubation with GEM (*P* < 0.01).

MHC-II, which is typically expressed on the surface of macrophages, monocytes and DCs, functions as a major regulator of immune responses by presenting antigens to CD4^+^ T cells [20]. We investigated whether GEM influenced the expression of MHC-II on M1 macrophages. The results showed that stimulation of RAW264.7 macrophages with IFN-γ/LPS led to high surface generation of MHC-II, which was markedly decreased when treated with GEM (Fig. 3C). As shown in Fig. 3D and E, western blot analysis indicated that GEM decreased the secretion of MHC-II in activated RAW264.7 cells (*P* < 0.05).

In summary, these results demonstrate that GEM reduces cytokine secretion and MHC-II expression in M1-type macrophages.

### Autophagy is downregulated in GEM-treated M1 macrophages

**Fig. 4:**
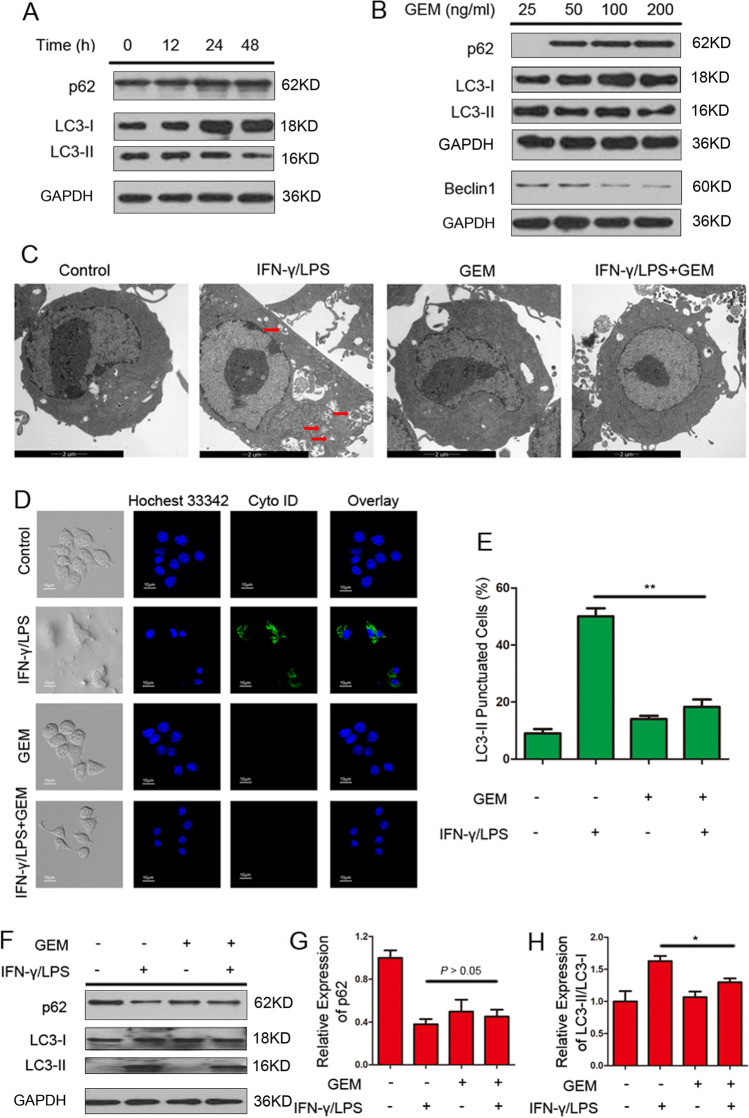
GEM-induced autophagy inhibition in RAW264.7 cells. **A** M1-activated RAW264.7 cells were exposed to 100 ng/ml of GEM for the indicated times, and then the expression of protein p62 and LC3-I/II were detected by western blot analysis. **B** M1-activated RAW264.7 cells were exposed to different concentrations of GEM (0–200 ng/ml) for 24 h, and then the expression of protein p62, LC3-I/II and Beclin1 were detected by western blot analysis. **C**–**F** Cells were treated with 300 IU/ml of IFN-γ and 100 ng/ml of LPS, either alone or in combination with 100 ng/ml of GEM for 24 h. **C** Autophagosomes in RAW264.7 cells were observed by TEM. **D** Confocal microscopy was used to detect the fluorescence of cells. **E** The quantitation analysis of fluorescent spots in Fig. 4B. **F** The expression of protein p62 and LC3-I/II were detected by western blot analysis. **G** Quantification of p62 was presented in bar graphs. **H** Quantification of LC3-II/LC3-I was presented in bar graphs. *P < 0.05, **P < 0.01

Evidence shows that autophagy acts as an immune responses regulator by reducing excessive inflammatory cytokine production, antigen presentation and intracellular pathogens [21]. Three well-established methods were employed to assess whether GEM influences the autophagic response of M1 macrophages [22]. First, we examined the expression of Sequestosome 1 (SQSTM1/p62) (an autophagic substrate) and the conversion of microtubule-associated protein 1 light chain 3 (LC3-I) to assess autophagy levels in RAW264.7 cells through western blot analysis. Figure 4A showed that the conversion of endogenous LC3-I to LC3-II in M1 macrophages was inhibited and the protein level of p62 was increased by GEM in time-dependent manner. Beclin 1 is known to initiate an autophagic process by forming an initial autophagosome complex [23]. The result indicated that GEM inhibited autophagy of activated macrophages in a dose-dependent manner (Fig. 4B). Second, the autophagic vacuoles presenting in macrophages were observed using TEM analysis. As shown in Fig. 4C, double membrane-enclosed autophagosomes were accumulated after IFN-γ/LPS stimulation of RAW264.7 cells; nevertheless, the quantity of autophagic vacuoles was decreased in GEM-treated cells (*P* < 0.01). Next, an autophagy detection kit (with green dye) was used to observe autophagic vacuoles. The results showed that RAW264.7 cells displayed more green fluorescence following stimulation with IFN-γ/LPS compared with untreated control cells. However, activated RAW264.7 cells incubated with GEM displayed limited green fluorescence (*P* < 0.05) (Fig. 4D and E). The macrophages treated with IFN-γ/LPS exhibited an obvious conversion of LC3-I to LC3-II and reduction of p62, whereas combination treatment with GEM-induced LC3-II conversion decreased and p62 levels increased in activated macrophages (Fig. 4F–H).

These data demonstrate that GEM inhibits the autophagosome formation of M1 macrophages.

### The role of autophagy in M1-activated macrophages

**Fig. 5:**
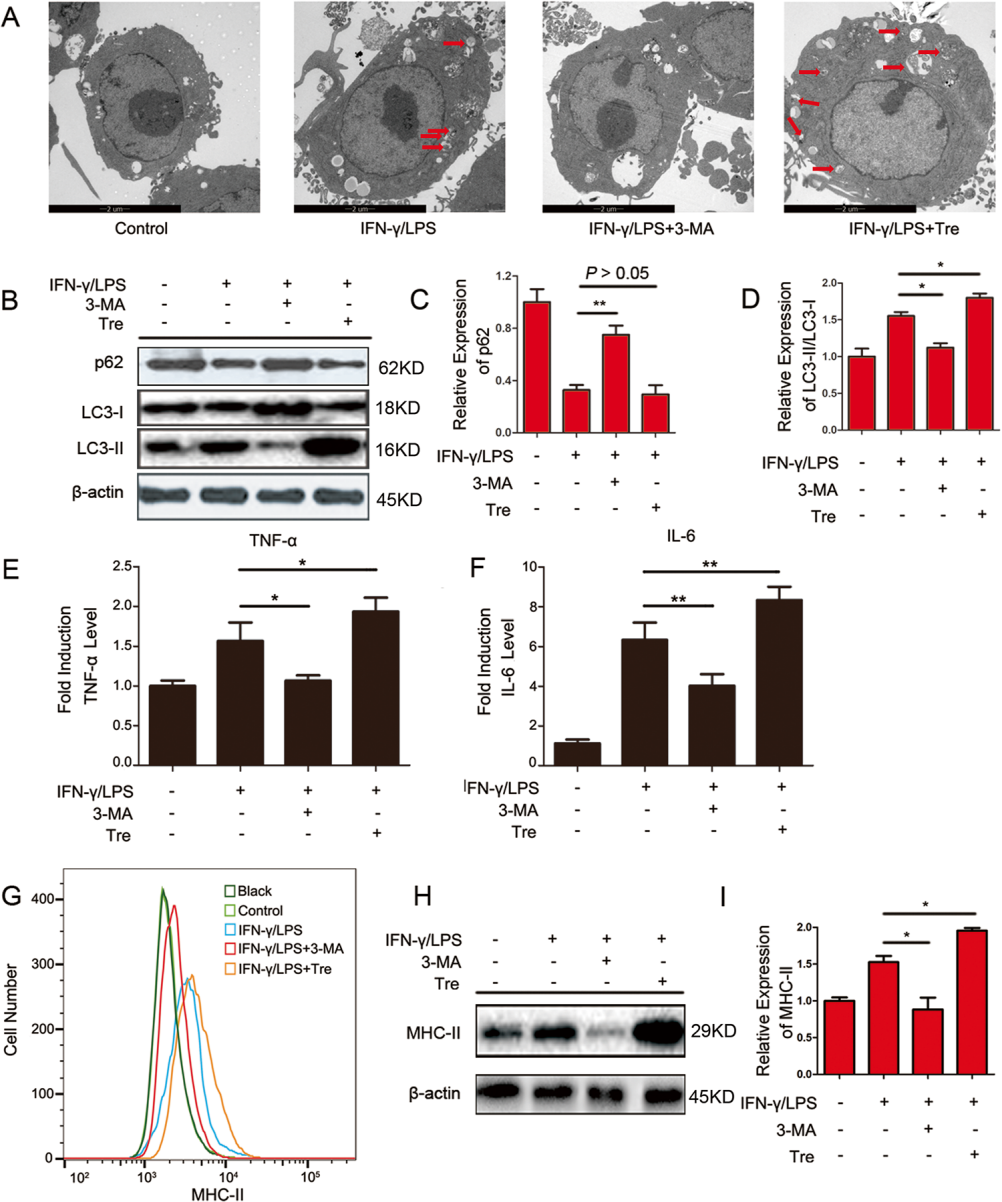
Immunoreactivity influenced by autophagy in RAW264.7 cells. Cells were treated with 300 IU/ml of IFN-γ and 100 ng/ml of LPS, either alone or in combination with 2 mM of 3-MA or 25 μM of Tre for 24 h. **A** Autophagosomes in RAW264.7 cells were observed by TEM. **B** The expression of protein p62 and LC3-I/II were detected by western blot analysis. **C** Quantification of p62 was presented in bar graphs. **D** Quantification of LC3-II/LC3-I was presented in bar graphs. **E** The content of TNF-α in the cell supernatant was detected by ELISA analysis. **F** The content of IL-6 in the cell supernatant was detected by ELISA analysis. **G** The MHC-II expression was measured by flow cytometry. **H** The expression of MHC-II was examined by western blot analysis. **I** Quantification of MHC-II was presented in bar graphs.*P < 0.05, **P < 0.01

The autophagy inhibitor 3-methyladenine (3-MA) and autophagy inducer Tre were used to investigate the function of autophagy in the production of TNF-α, IL-6 and MHC-II in M1 macrophages. The results of TEM and western blot showed that 3-MA effectively inhibited autophagy and Tre activated autophagy in M1-activated macrophages (*P* < 0.05) (Fig. 5A–D). Following incubation of M1-activated macrophages with Tre, the levels of TNF-α and IL-6 were significantly increased. In contrast, TNF-α and IL-6 levels were apparently decreased after incubation of M1 macrophages with 3-MA (*P* < 0.05) (Fig. 5E and F). However, flow cytometry and western blot analyses indicated that MHC-II surface expression in M1-activated macrophages was decreased when treated with 3-MA (*P* < 0.05) and was increased when treated with Tre (*P* < 0.05) (Fig. 5G–I). The above results indicate that autophagy plays an important role in M1-activated macrophages.

### Autophagy is involved in GEM-induced immune inhibition of M1-activated macrophages

**Fig. 6:**
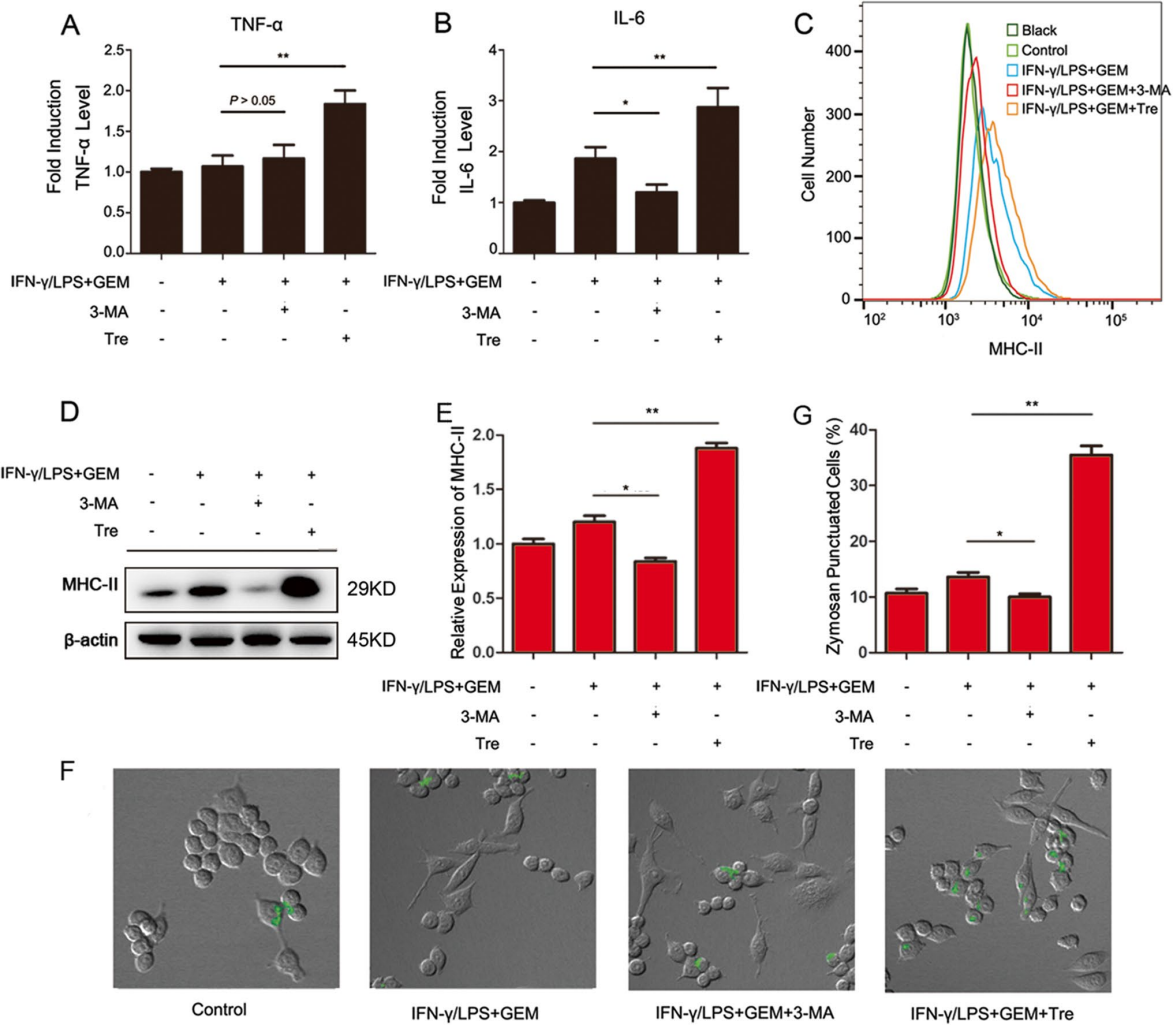
The role of autophagy in GEM-induced immune inhibition in RAW264.7 cells. Cells were treated with 300 IU/ml of IFN-γ, 100 ng/ml of LPS and 100 ng/ml of GEM, either alone or in combination with 2 mM of 3 MA or 25 μM of Tre for 24 h. **A** The content of TNF-α in the cell supernatant was detected by ELISA analysis. **B** The content of IL-6 in the cell supernatant was detected by ELISA analysis. **C** The MHC-II expression was measured by flow cytometry. **D** The expression of MHC-II was examined by western blot analysis. **E** Quantification of MHC-II was presented in bar graphs. **F** Macrophages were treated with 2 mg/ml of zymosan particles for another 2 h and then observed via confocal microscopy. **G** The quantitation analysis of fluorescent spots in Fig. 6F. *P < 0.05, **P < 0.01

Based on these findings, we examined the role of autophagy on TNF-α and IL-6 secretion, MHC-II expression and phagocytosis in GEM-treated M1-activated macrophages. As shown in Fig. 6A and B, after incubation of GEM-treated M1-activated macrophages with 3-MA, there were no obvious changes in the secretion of TNF-α, but the generation of IL-6 was obviously lower compared with the levels in GEM-treated cells. In contrast, the productions of TNF-α and IL-6 were all increased in GEM-treated M1 macrophages after incubation with Tre. Flow cytometry and western blot analyses indicated that MHC-II surface expression in GEM treated M1-activated macrophages was decreased when treated with 3-MA (*P* < 0.05) and increased when treated with Tre (*P* < 0.01) (Fig. 6C–E). Figure 6F–G and Fig. S4 showed that 3-MA could inhibit the phagocytosis of GEM-treated M1 macrophages, and Tre promoted the phagocytosis of GEM-treated M1 macrophages. These results show that upregulation of autophagy can block GEM-induced immune inhibition in M1-activated macrophages.

## Discussion

Previous researches have reported that GEM treatment promotes an immunosuppressive microenvironment in PAC tumours by supporting the migration, invasion, growth and polarization of murine macrophages [24]. GEM was shown to reduce the levels of inflammatory cytokines, such as monocyte chemotactic protein-1 (MCP-1), IL-12p40, eotaxin, macrophage inflammatory protein-1 beta (MIP-1β) and TNF-α [25]. GEM treatment also decreased immune-suppressive myeloid-derived suppressor cells and increased immune-stimulating M1 macrophages [26]. M1 macrophages stimulate the release of cytokines, such as TNF-α, IL-6, IL-1β and IL-12, which results in the activation of naïve recipient immune cells [27]. Until now, the effects of GEM on M1 macrophage functions and the underlying molecular mechanism remain unknown.

INF-γ is defined as an immune-activating cytokine for macrophages in mice [28, 29]. It has been confirmed that macrophages can be generated from monocytes in vitro and undergo classical (LPS + IFN-γ treated) activation. In vitro classically activated (LPS + IFN-γ) is widely considered synonymous with in vivo M1 [30]. In this study, we used IFN-γ/LPS to stimulate RAW264.7 cells to generate M1 macrophages [14] and assessed the activities and functions of these cells, including cell proliferation, cell cycle status, phagocytosis, free radical release, the secretion of pro-inflammatory cytokine and the expression of MHC-II expression after GEM treatment. We observed that GEM not only induced G_0_/G_1_ arrest to prevent cells from proliferating but also impaired the phagocytic function of macrophages. Classically activated M1 macrophages are typically characterized by increased expression of ROS and nitrogen intermediates which can enhance the microbial killing ability of macrophage [31]. Confocal microscopy with a ROS-detecting dye and a Griess reagent assay showed that GEM inhibited the secretion of ROS and NO, respectively, in IFN-γ/LPS-treated macrophages. Besides secretion of superoxide, activated macrophages are also featured by its high microbicidal function associated with the ability to generate high levels of cytokines such as TNF-α, IL-6 and IL-1β [32]. MHC-II molecules are primarily expressed by professional antigen-presenting cells such as B cells, dendritic cells and macrophages. In a range of human tumours and murine models, MHC-II expression and regulation is considered as critical step with presentation of extracellular pathogens to T helper cell [33]. The phagocytosis of activated macrophages plays a critical role in the first line of microorganism defense in both adaptive and innate immunity. Our data showed that GEM hampered TNF-α and IL-6 cytokines secretion, MHC-II expression and phagocytosis in activated macrophages.

The current findings identified the immunosuppressive effect of GEM on macrophages; however, the mechanism was poorly revealed. Accumulating evidence has shown that autophagy plays crucial roles in regulating innate and adaptive immunity and inflammatory responses [16, 34]. Therefore, the effect of autophagy in GEM-induced immune inhibition of M1 macrophages was investigated. Three well-established approaches were used to assess autophagosome formation in RAW264.7 macrophages. It has been confirmed that GEM inhibited the autophagic response in M1 macrophages, as evidenced by the downregulation of autophagosomes via TEM analysis, the reduced formation of autophagosome-like vacuoles by confocal microscopy analysis and lower expression of autophagy-related protein LC3-II by western blot analysis.

Based on the above results, GEM not only inhibited the function of immunity but also suppressed cell autophagy in M1 macrophages. As GEM is employed as anti-cancer therapeutics in clinic, it is important to investigate the possibility to increase macrophage immune response. Here, M1 macrophages were treated with autophagy inhibitor 3-MA and autophagy inducer Tre. The data demonstrated that the formation of autophagosomes, the secretion of IL-6 and TNF-α and the expression of MHC-II were significantly decreased when autophagy was inhibited in M1 macrophages. The opposite results were observed following treatment with Tre. Subsequently, activated macrophages were treated with 3-MA or Tre in combination with GEM. The results showed that overcoming GEM-induced autophagy suppression with Tre could restore TNF-α and IL-6 secretion, MHC-II expression and phagocytosis capacity in M1 macrophages. These findings suggested that Tre could successfully restore immune inhibition induced by GEM of activated macrophages.

In summary, this study demonstrated that GEM impaired the activities and functions of macrophages, including cell cycle, phagocytosis, free radical and cytokine secretion and MHC-II expression, and therefore induces immune inhibition of macrophages. Further research indicated that activating autophagy obviously ameliorated GEM-induced immune inhibition in activated macrophages. GEM suppressed the function of macrophages via autophagy-mediated mechanism. However, murine monocytes applied in this study were a little weak to confirm our findings. We will use primary immune cells as models to further explore the functions and mechanisms in GEM-treated immune cells in our future research.

## Supplementary Information

Below is the link to the electronic supplementary material.

Supplementary file1 (DOCX 5314 kb)

## Abbreviations

3-MA: 3-Methyladenine
GEM: Gemcitabine
IFN-γ: Interferon-γ
IL-6: Interleukin 6
LPS: Lipopolysaccharide
MCP-1: Monocyte chemotactic protein-1
MHC-II: Major histocompatibility complex II
NO: Nitric oxide
OD: Optical density
PBS: Phosphate-buffered saline
ROS: Reactive oxygen species
TAMs: Tumour-associated macrophages
TEM: Transmission electron microscopy
TNF-α: Tumour necrosis factor α
Tre: Trehalose
