# Supplementary material for: Activation of autophagy reverses gemcitabine-induced immune inhibition of RAW264.7 macrophages by promoting TNF-α, IL-6 and MHC-II expression
Source: Immunol Res. 2021 Jul 14;69(4):352–62. doi: 10.1007/s12026-021-09210-7 (PMC8342359; doi:10.1007/s12026-021-09210-7)
Supplement: Supplementary file 1 — Supplementary file1 (DOCX 5314 kb) [file 12026_2021_9210_MOESM1_ESM.docx]

**Activation of Autophagy Reverses Gemcitabine-induced Immune Inhibition of RAW264.7 Macrophages by promoting TNF-α, IL-6 and MHC-II expression**

**Shanshan Jiang^1^*·Rong Wang^1^*·Lu Han^1*^·Kudelaidi Kuerban^2^·Li Ye^2^·Shu Pan^1^·Shengnan Li^1^·Yongfang Yuan^1^**

^1^ Department of Pharmacy, Shanghai Ninth People’s Hospital, Shanghai JiaoTong University School of Medicine, Shanghai 200011, China

^2^ Department of Microbiological and Biochemical Pharmacy & The Key Laboratory of Smart Drug Delivery, Ministry of Education, School of Pharmacy, Fudan University, Shanghai 201203, China

*These authors contributed equally to this work.

🖂 Yongfang Yuan:

Phone: +86 21 5678 6907

Fax: +86 21 5678 6907

E-mail: nmxyyf@126.com

**Supplementary Figures**


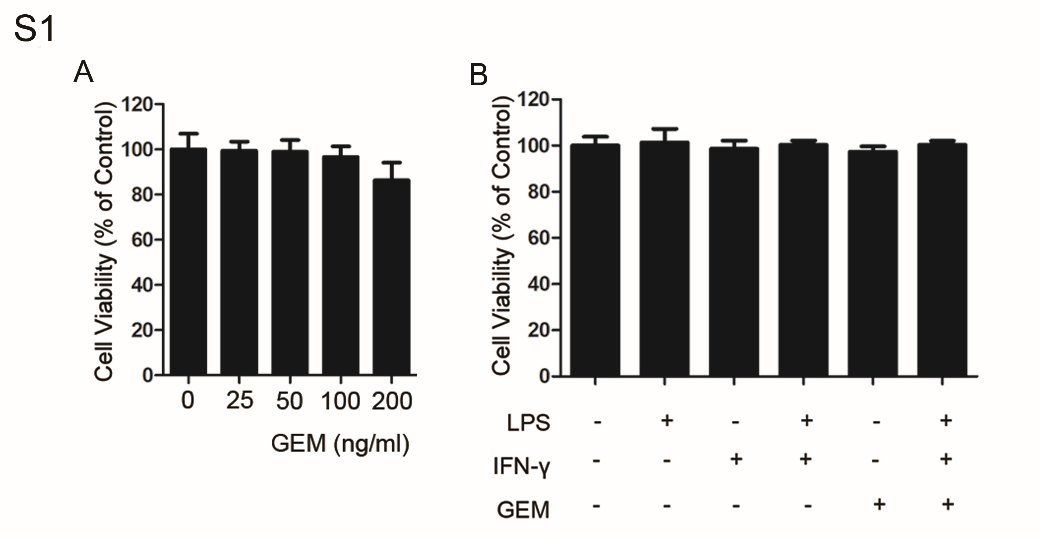


Fig.S1 Cytotoxic effect of LPS, IFN-γ and GEM on RAW264.7 cells. A. RAW 264.7 cells were treated with different concentrations of GEM (0-1000 ng/ml) for 24 h. Cell viability was detected by MTT assay. B. RAW264.7 cells were incubated with 300 IU/ml of IFN-γ or 100 ng/ml of LPS, with or without 100 ng/ml of GEM for 24 h. The cell viability was detected by MTT assay. ***P* < 0.01 versus control, **P* < 0.05 versus control.


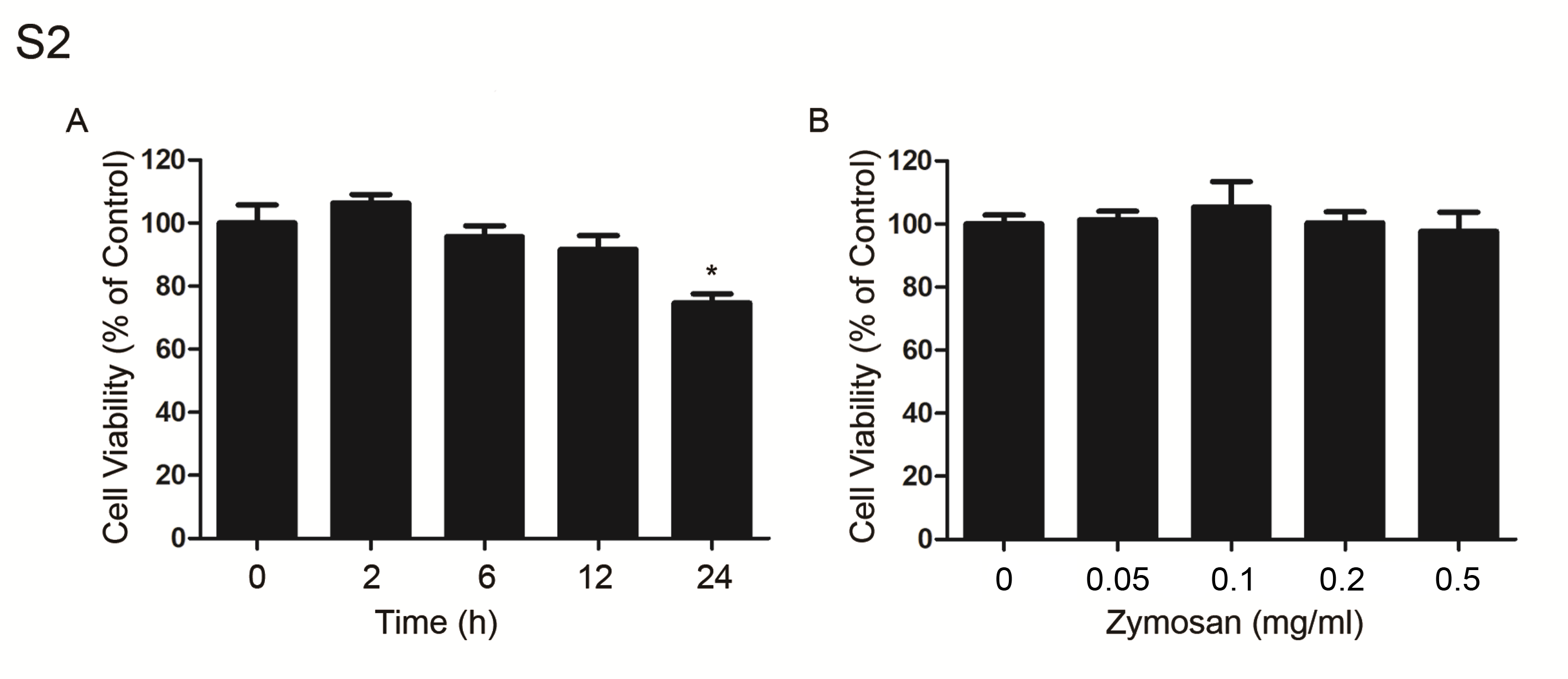


Fig.S2 The cytotoxic of zymosan on RAW264.7 cells. A. RAW264.7 cells were treated with 100 μg/ml of zymosan for different times. The cell viability was detected by MTT assay. B. RAW264.7 cells were treated with different concentrations of zymosan for 2 h. The cell viability was detected by MTT assay. **P* < 0.05 versus control.


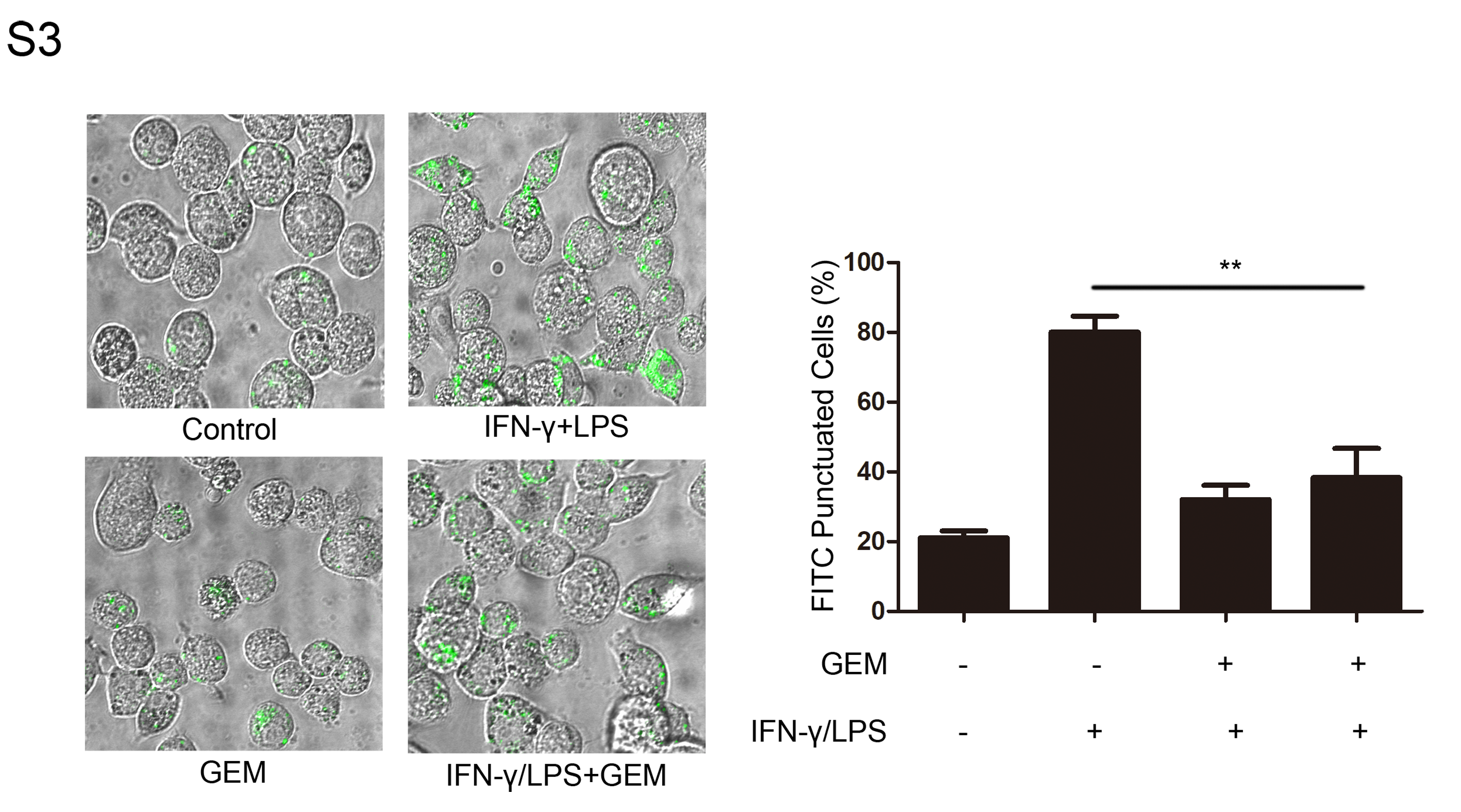


Fig.S3 Phagocytosis is inhibited by GEM in RAW264.7 cells. Cells were treated with 300 IU/ml of IFN-γ and 100 ng/ml of LPS, either alone or in combination with 100 ng/ml of GEM for 24 h. RAW264.7 cells were treated with FITC-labeled *E.coli* *DH 5α* for another 2 h and the fluorescent punctuation was observed through confocal microscopy. ***P* < 0.01.


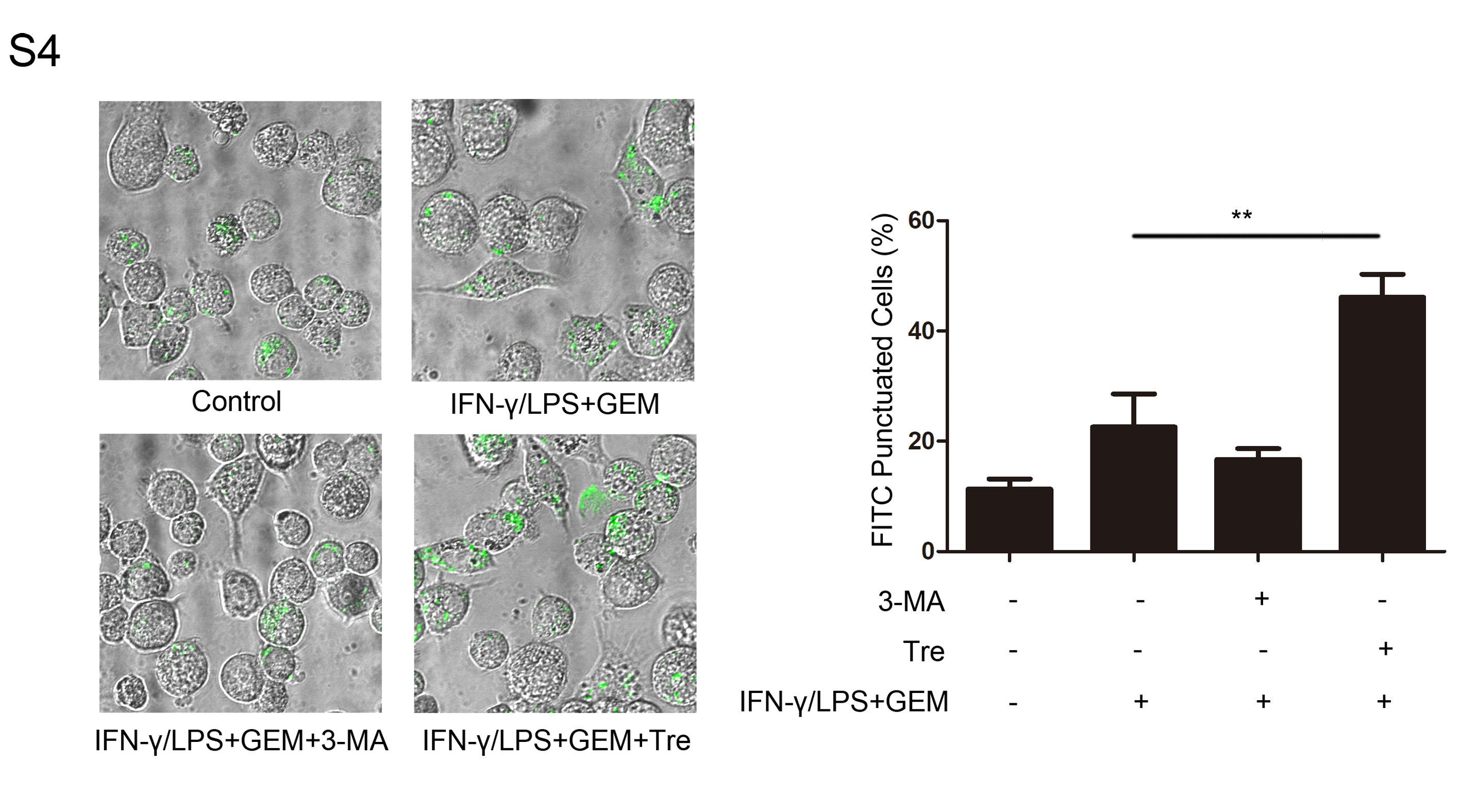


Fig.S4 The role of autophagy in GEM-induced phagocytosis inhibition in RAW264.7 cells. Cells were treated with 300 IU/ml of IFN-γ, 100 ng/ml of LPS and 100 ng/ml of GEM, either alone or in combination with 2mM of 3MA or 25 μM of Tre for 24 h. And then, RAW264.7 cells were treated with FITC-labeled *E.coli DH 5α* for another 2 h and the fluorescent punctuation was observed through confocal microscopy. ***P* < 0.01.
